# A Novel Triple Combination Therapy for the Treatment of Recalcitrant Plantar Warts: A Case Report

**DOI:** 10.7759/cureus.70680

**Published:** 2024-10-02

**Authors:** Mohammed Albeshri

**Affiliations:** 1 Dermatology, College of Medicine, Qassim University, Buraydah, SAU

**Keywords:** cryotherapy, dermatology, imiquimod, intralesional injection of purified protein derivative (ppd), plantar wart

## Abstract

Recalcitrant plantar warts, caused by human papillomavirus, often resist conventional treatments, necessitating alternative approaches. This case report details the successful treatment of a persistent plantar wart in a 24-year-old male using a novel triple combination therapy. The patient had previously undergone multiple unsuccessful treatments, including cryotherapy, salicylic acid, and duct tape occlusion. The combination therapy consisted of cryotherapy, topical imiquimod 5% cream, and intralesional injections of purified protein derivative (PPD). The regimen was administered over six weeks, with cryotherapy and PPD injections every three weeks, and imiquimod applied every other day. Significant regression was observed by the second session, with near-complete resolution by the third. At six-month follow-up, no recurrence was noted, and the skin had healed without scarring. This case highlights the synergistic effects of combining physical destruction (cryotherapy), immune modulation (imiquimod), and local immune stimulation (PPD) to successfully treat recalcitrant plantar warts. This triple combination therapy may offer a promising option for cases unresponsive to standard treatments, providing a comprehensive approach that reduces recurrence and promotes complete resolution.

## Introduction

Warts are the most common cutaneous manifestation of human papillomavirus (HPV), which infects both skin and mucosal epithelial tissues. While warts are typically self-limiting in immunocompetent individuals, they can become recalcitrant, prompting patients to seek treatment due to discomfort, pain, or social stigma, particularly with plantar warts [1,2]. Recurrence rates after treatment are notably high, with around 30% relapsing, often due to viral reservoirs in the surrounding tissue. Traditional destructive methods, such as cryotherapy and electrocautery, are effective but prone to recurrence, as they may not fully eradicate HPV or modulate the immune response [3].

Immunotherapeutic treatments - whether topical, intralesional, or systemic - are gaining popularity in wart management due to their nondestructive nature, safety, suitability for pediatric patients, and lower recurrence rates [4,5]. These therapies, including intralesional immunotherapy with antigens like *Candida *or purified protein derivative (PPD), stimulate the host’s immune response, particularly cell-mediated immunity, which involves Th1 cytokine production to eliminate HPV infection [6]. Imiquimod, an immune response modifier that activates TLR7, is also effective for treating HPV-related warts by enhancing the immune system’s activity [7].

In this case report, we present the successful and safe treatment of a recalcitrant plantar wart using a triple combination therapy involving intralesional PPD, imiquimod cream, and cryotherapy. This multimodal approach effectively resolved the persistent lesion, offering a promising strategy for managing difficult-to-treat wart cases.

## Case presentation

**Figure 1:**
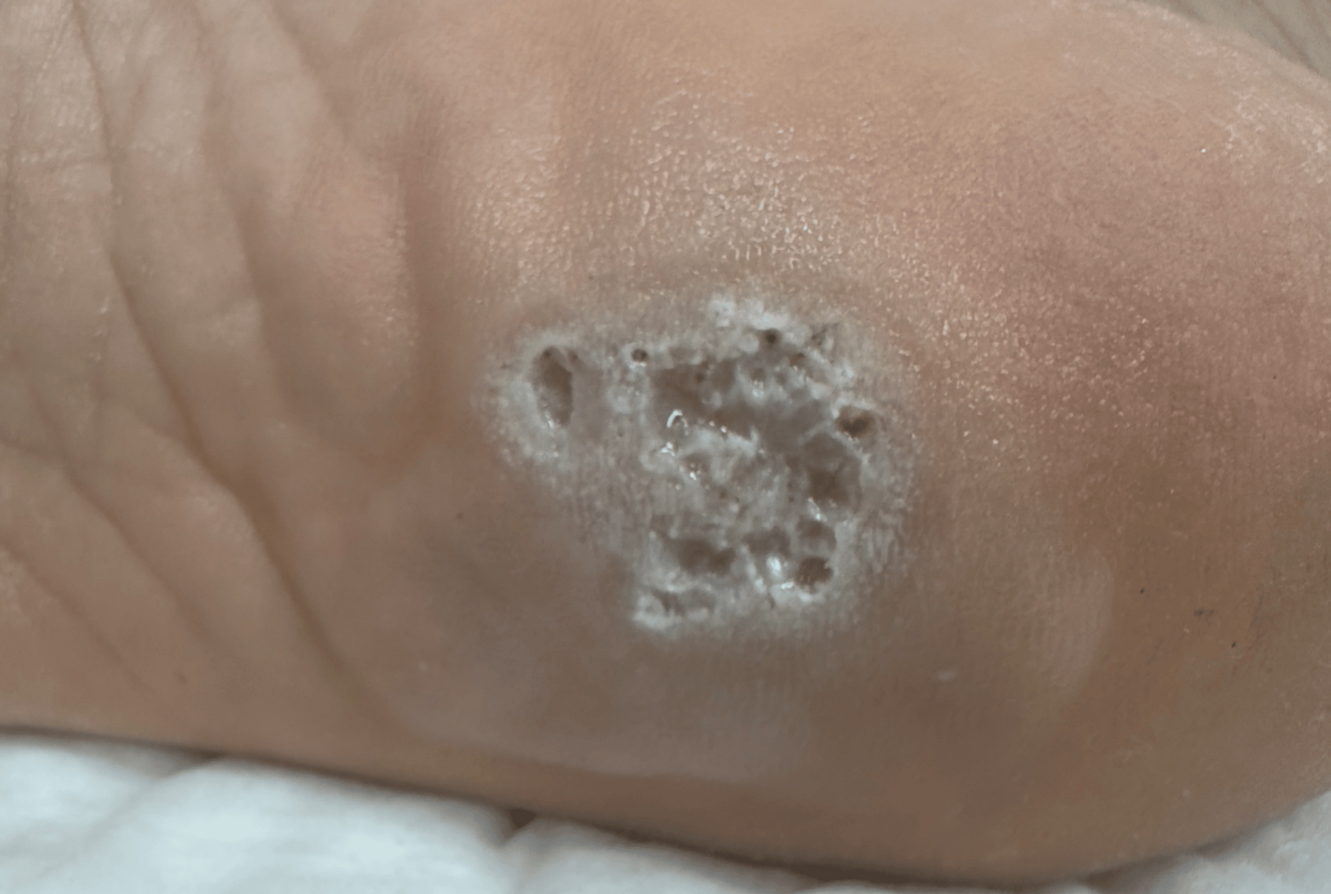
Pre-treatment appearance of the wart

A 24-year-old male with a recalcitrant plantar wart on his left foot experienced persistent pain and had undergone multiple failed therapies for over a year, including salicylic acid solution, traditional duct tape, and cryotherapy alone (Figure 1). He underwent a multimodal treatment approach that combined cryotherapy, intralesional PPD, and topical imiquimod 5% cream. The initial treatment involved cryotherapy to freeze and destroy the wart, followed by an intralesional injection of 0.01 ml PPD to stimulate a localized immune response. The patient was instructed to apply imiquimod every other night to enhance immune activation further.

**Figure 2:**
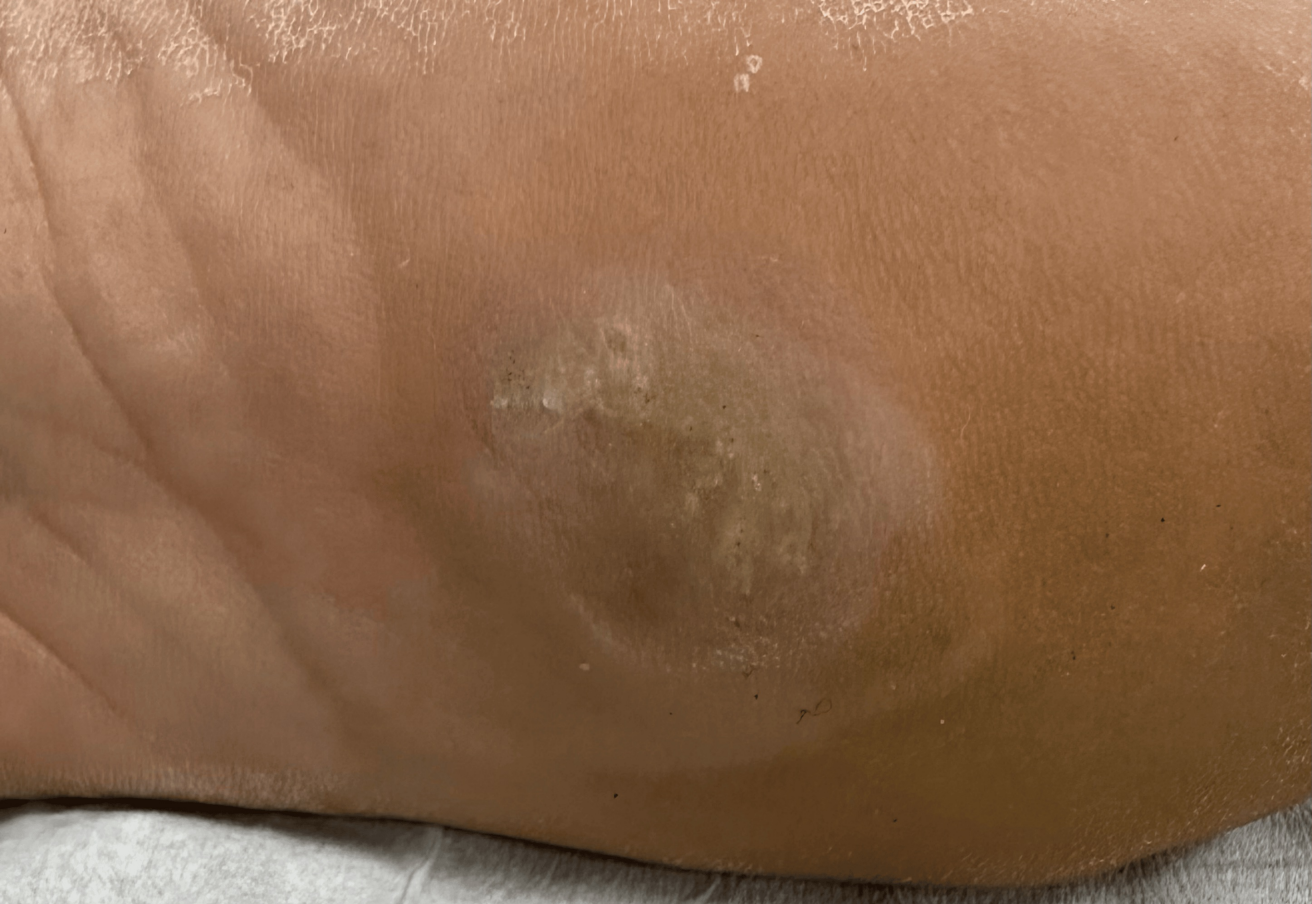
Appearance of the plantar wart after the first treatment session

**Figure 3:**
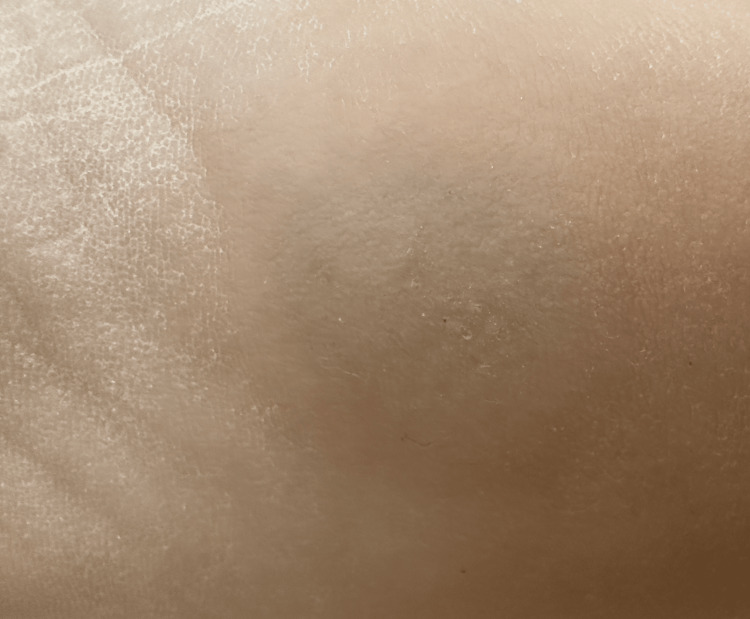
Near-complete resolution of the plantar wart following the third treatment session

Three weeks after the initial treatment, the patient returned for a second session, during which another round of cryotherapy and an intralesional PPD injection were administered, and he continued applying the imiquimod 5% cream. At this stage, signs of regression were evident, with the wart reducing in size and thickness, indicating that the combination of direct wart destruction and immune enhancement was effective (Figure 2). By the sixth week, after a third session of cryotherapy and PPD injection, the wart had almost completely resolved, and the surrounding skin exhibited significant healing with minimal scarring or adverse effects (Figure 3). The continued use of imiquimod during the treatment played a critical role in sustaining the immune response, further reducing the viral load and preventing the reestablishment of the infection.

The combination of cryotherapy, imiquimod, and intralesional PPD led to the complete resolution of the plantar wart, as shown in Figure 3. The patient was followed up for six months, and no recurrence was noted. The skin healed without scarring.

## Discussion

The success of the combination therapy involving PPD and topical imiquimod 5% cream underscores the potential of a multimodal approach in treating recalcitrant plantar warts. Cryotherapy offers immediate physical destruction of wart tissue, while imiquimod enhances the immune response. Imiquimod is primarily used for viral skin infections, such as genital warts, and also for other skin conditions like actinic keratosis and superficial basal cell carcinoma. Its off-label use for treating stubborn cutaneous warts, including plantar warts, is due to its ability to enhance the body’s immune response and induce localized inflammation [7,8].

Imiquimod stimulates the immune system to produce cytokines, such as interferon-alpha, tumor necrosis factor, and interleukin-12. These cytokines activate immune cells, including macrophages and T-cells, which target and eliminate virus-infected cells, leading to wart resolution [8].

Intralesional PPD further stimulates local immunity. Commonly used in tuberculosis skin testing, PPD serves as an immunotherapeutic option for recalcitrant warts, including plantar warts. This technique triggers a localized immune response to help the body fight the HPV that causes warts [9].

PPD injections act as an immune stimulus, mimicking an infection to provoke the immune system’s response. This leads to increased production of cytokines such as interferon and tumor necrosis factor, which not only suppress and control the wart but also activate immune cells that recognize and destroy HPV-infected cells. This novel use of PPD effectively leverages the body’s immune system to target and eradicate persistent wart infections, presenting a promising option for individuals with recalcitrant warts [10].

A review of 15 studies conducted between 1984 and 2020 assessed the efficacy of cryotherapy in 600 patients, revealing variable cure rates ranging from 0% to 100%, with an average cure rate of 45.61% [11,12]. The mean follow-up period for cryotherapy was 14.7 weeks, and the number of sessions ranged from 1 to 12. Additional studies published between 1997 and 2020, focusing on physical treatments like photodynamic or laser therapy in 236 patients, reported an average cure rate of 79.36%, with a range of 53% to 96%, a mean follow-up period of 36 weeks, and up to seven sessions. Ultrasound therapy showed an 81% cure rate, while hyperthermia had a 53.57% cure rate [13-15].

The review of intralesional and topical immunotherapies for plantar warts highlights their variable effectiveness. Intralesional immunotherapy, including injections of vitamin D and antigens like *Candida*, showed cure rates between 25% and 87%, possibly due to differences in antigens used and patient immune responses. Topical immunotherapy demonstrated a lower cure rate of 34%, likely due to limitations in skin penetration or localized immune activation [16]. Additionally, Falknor’s multipuncture method, designed to stimulate the immune response, achieved only a 14% cure rate after one session. This variability underscores the importance of optimizing treatment protocols and combining therapies to enhance outcomes, especially given the diverse immune responses among patients [17].

## Conclusions

This case demonstrates the successful treatment of resistant plantar warts through a combination of cryotherapy and immune-modulating agents, PPD and imiquimod. PPD stimulates local immune responses, while imiquimod enhances the body’s HPV-targeted defense. This dual-action approach effectively resolved the wart, minimized recurrence, and prevented scarring, which is commonly associated with more destructive treatments. The combination therapy provides a promising alternative for managing persistent HPV-related lesions in dermatology.
